# Tubular Ileal Duplication Causing Perforative Peritonitis in an Adult: A Rare Presentation

**DOI:** 10.1055/s-0040-1722180

**Published:** 2021-02-01

**Authors:** Jignesh A. Gandhi, Pravin H. Shinde, Jayati J. Churiwala, Sakina Husain

**Affiliations:** 1 Department of Surgery, Seth G.S. Medical College & K.E.M. Hospital, Mumbai, Maharashtra, India

**Keywords:** enterogenous cyst, enteric duplication cyst, duplication cyst, ileal duplication, tubular duplication, perforative peritonitis

## Abstract

**Background** Enteric duplication cysts are a rare congenital abnormality that present more commonly in children than adults. Clinical presentation varies from vague abdominal pain, abdominal lump, iron deficiency anemia to intestinal obstruction due to intussusception or mass effect. We report a tubular ileal duplication in an adult male presenting with an acute abdomen due to perforative peritonitis.

**Case** A 20-year-old male presented to the emergency department with complains of right lower abdominal pain. On clinical examination and ultrasound scan patient was suspected to have a complicated acute appendicitis (rupture). However, a computed tomography scan was suggestive of perforation in the distal ileum. Emergency exploratory laparotomy revealed a perforated isolated ileal tubular duplication.

Enteric duplication cysts are a rare congenital abnormality that present more commonly in children than adults. 1 Clinical presentation varies according to the site and size of the cyst. Although they may arise from any part of the alimentary tract, their origin is most frequently from the small bowel (jejunum more than ileum) in the form of cystic or tubular conformation which may be continuous with or adherent to the bowel wall or mesentery. 2 3 4 Gastroenteric duplication cysts present with diverse features ranging from vague abdominal pain, abdominal lump, iron deficiency anemia to intestinal obstruction due to intussusception or mass effect. 5 6 Symptoms may mimic a perforated Meckel's diverticulum in cysts lined by heterotopic gastric mucosa. Surgical management of these patients is determined by their mode of presentation. Cysts continuous with the lumen of the adjacent bowel require resection of the same segment of bowel along with the cyst, while those which are totally isolated demand only cyst excision. We report a tubular ileal duplication in an adult male presenting with an acute abdomen due to perforative peritonitis.

## Case Report

**Fig. 1:**
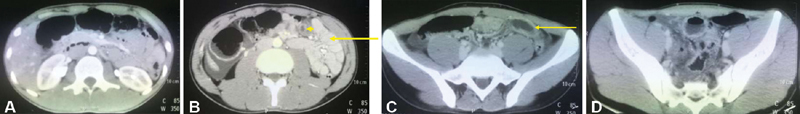
Axial sections of Contrast enhanced computed tomography (CECT) scan of the abdomen and pelvis (intravenous and oral contrast) ( **A** ) showing pneumoperitoneum. ( **B** ) the ileal duplication cyst (short arrow) did not take up oral contrast (compare with ileal loops marked with long arrow) suggestive of lack of communication with the gastrointertinal tract. ( **C** ) the ileal duplication cyst (arrow) did not take up oral contrast (as in **B** ). ( **D** ) minimal free fluid in the pelvis tracking down from the right iliac fossa.

A 20-year-old male patient presented to the emergency department of a tertiary care center with complains of intermittent, vague, dull aching pain in the abdomen localized to the periumbilical region for 2 years managed conservatively with medications from a local practitioner aggravated since 1 day, now localized to the right lower abdomen and associated with one episode of nonbilious vomiting. There was no history of per rectal bleed, melena, or loss of weight and appetite. On clinical examination the patient had tachycardia. Abdominal examination revealed tenderness and guarding in the right iliac fossa. A clinical diagnosis of acute appendicitis was made. An erect abdominal X-ray did not show free gas under the right dome of diaphragm. An ultrasound scan of the abdomen was reported as acute appendicitis with suspected rupture with free fluid in the pelvis. A computed tomography scan of the abdomen and pelvis was obtained. This was suggestive of a thickened segment of fluid-filled distal ileum with no intraluminal oral contrast, with surrounding free fluid and extraluminal air specks in addition to reactive mesenteric lymphadenopathy with minimal free fluid in the pelvis tracking down from the right iliac fossa ( Fig. 1A–D ). The appendix appeared normal. The patient underwent emergency exploratory laparotomy for perforative peritonitis.

**Fig. 2:**
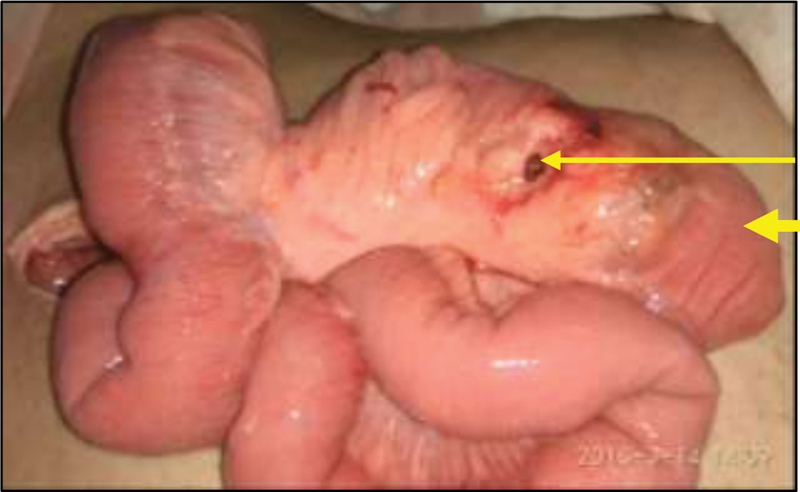
Intraoperative picture. Perforation (thin arrow) on the mesenteric aspect of the tubular ileal duplication (thick arrow).

**Fig. 3:**
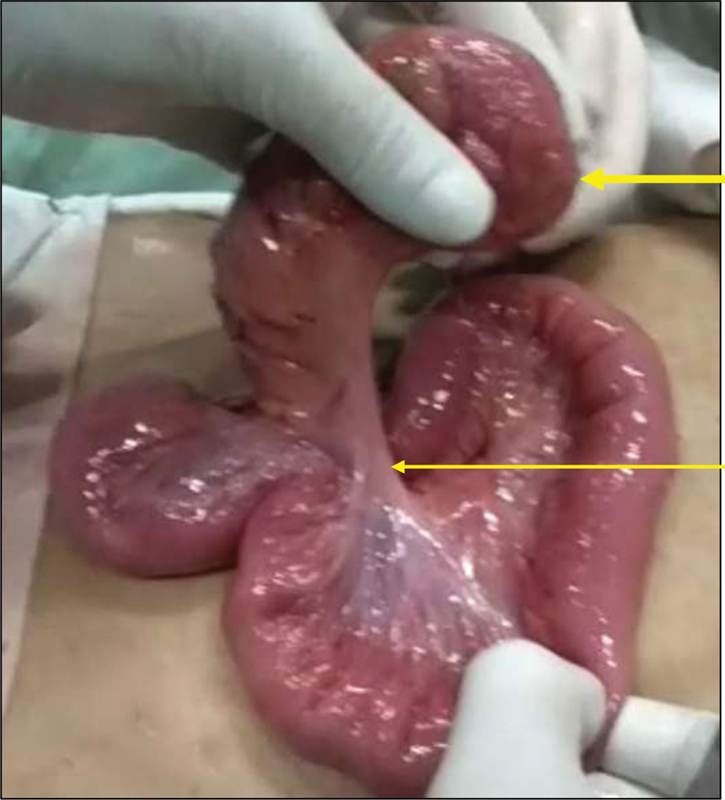
Intraoperative picture. Mesenteric pedicle (thin arrow) to the isolated ileal duplication (thick arrow).

**Fig. 4:**
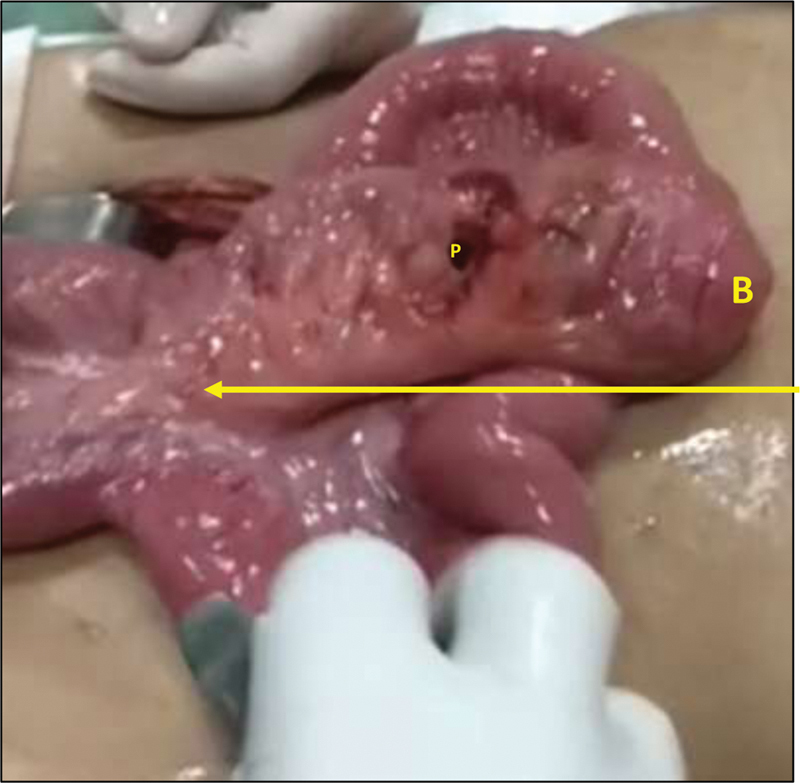
Mesenteric pedicle (arrow) to the isolated tubular ileal duplication arising from the ileal mesentery. B, blind end; P, perforation.

A 15-cm long isolated tubular ileal duplication ( Fig. 2 ), 30 cm proximal to the ileocecal junction supplied by a separate mesenteric pedicle arising from the base of the ileal mesentery ( Figs. 3 and 4 ) with a 1-cm wide perforation on the mesenteric aspect of the duplication was found. There was 200 mL mucoid contamination of the peritoneal cavity. Rest of the small and large bowel was normal.

**Fig. 5:**
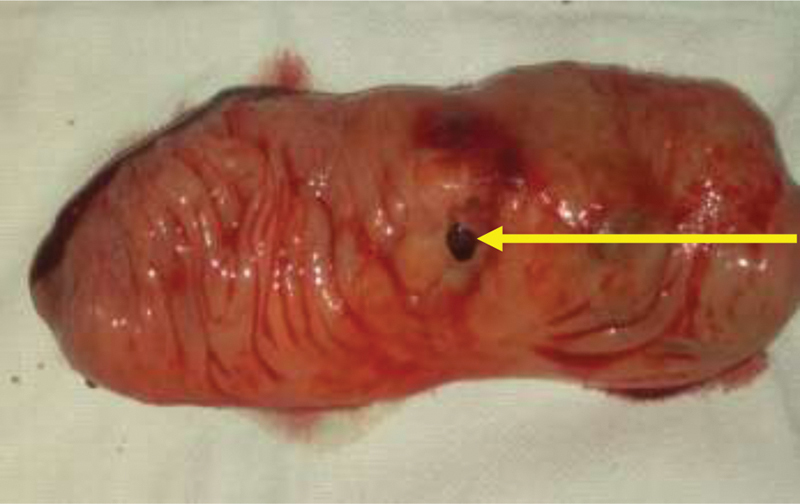
Resected ileal duplication with perforation on the mesenteric aspect (arrow).

The ileal duplication was resected along with its mesenteric pedicle ( Fig. 5 ). After a thorough peritoneal lavage, the abdomen was closed. We did not place any drains in the abdomen. He was ambulatory and the nasogastric tube was removed on the first postoperative day (POD) and the patient started on oral sips. Once tolerated, he was progressed to liquids and then soft diet on POD 3. He was discharged on POD 4. Patient was followed up in the outpatient department on POD 10 for suture removal. He had an uneventful postoperative recovery.

Histopathology report confirmed an ileal duplication cyst lined by gastric mucosa with foveolar hyperplasia and chronic gastritis.

## Discussion

Gastrointestinal (GI) duplication cysts are rare congenital anomalies that have been classified according to origin as foregut, midgut, and hindgut duplication. They most commonly originate from the small bowel and can be cystic or tubular in morphology. 7 Spherical or cystic duplications are more common than tubular ones. 8 Li et al have further classified small bowel duplications into type I and II depending on the vascular supply pattern to the duplication and the adjacent bowel. 9 Several theories have been proposed for the origin of duplication cysts like partial or abortive twinning, intrauterine vascular accidents, diverticular and vacuolization defects, and the most widely accepted split notochord theory.

Enteric duplications can arise from the submucosal, intermuscular, or subserosal layer of the bowel wall which determines the attachment, continuity, and detachment of the duplication from the bowel wall. 10 For instance, duplications arising from the subserosal layer may detach from the bowel wall and lie adjacent to the point of its origin. In our case, we report an isolated type I tubular ileal duplication.

Duplication cysts are usually diagnosed incidentally on an antenatal or perinatal ultrasound scan. 11 They may present as a slowly growing abdominal lump in children in the first decade of life as reported by Srivastava et al. 12 Primary presentation in adults is a rare occurrence. When presenting after the second decade of life, the clinical picture varies from abdominal pain, abdominal lump, hematochezia (small and large bowel duplication), melena (foregut duplication), small bowel obstruction, intussusception, volvulus, and rarely malignancy in the duplication cyst. 5 Although it has been suggested that peptic ulcer in a duplication cyst due to acid secreting heterotopic gastric mucosa may lead to perforative peritonitis, on our literature search, we have come across few such case reports in world literature.

Recurrent abdominal pain, as in our patient, may be one of the presentations of an enteric duplication cyst. Akin to a mucocele of the appendix, distension of an isolated cyst (not communicating with the bowel lumen) with secretions from the lining mucosa may be the source of abdominal pain. We, therefore, emphasize the need to differentiate these two conditions which may present in a similar age group. Of particular interest, however, is the peritonism caused by the secretions on perforation of the cyst in the absence of biliary or feculent contamination of the peritoneal cavity. This presentation due to chemical peritonitis caused by acid production from the lining gastric mucosa mandates a thorough search for a duplication cyst because lack of communication with the bowel lumen makes pneumoperitoneum an unlikely finding on radiological investigation. As a corollary, bacterial contamination of the free fluid by translocation leading to secondary peritonitis may be a late presentation. Infrequently, it may be attributed to a sealed off bowel perforation due to a similar clinical and radiological presentation.

Mucinous secretions from a perforated cyst, on the other hand, may mimic pseudomyxoma peritonei and come to notice only on secondary infection leading to peritonitis or on diagnostic laparoscopy for evaluation of abdominal pain. As reported by Lemahieu et al, a 67-year-old woman who presented with vague abdominal pain for a duration of 6 months was diagnosed with disseminated peritoneal adenomucinosis on exploratory laparoscopy secondary to leak from/rupture of an intestinal duplication with a low grade villous adenoma discovered on subsequent surgery. 13 A thorough search for an enteric duplication in the absence of another source of peritonitis or pseudomyxoma-like picture, may thus be rewarding.

In 1984, a 21-year-old French male patient reportedly suffering from 4 years of abdominal pain with recurrent GI bleed and spontaneously resolving peritonitis was diagnosed during a second laparotomy on a 99m pertechnetate scan to have ectopic gastric mucosal ulceration in a tumescent segment of terminal ileum which on histopathology was a duplication cyst. 14 Small and tubular duplications are difficult to diagnose on preoperative radiological scans in the emergency setting. Given the rarity of their occurrence in adults, a vigilant search for this entity in patients with chronic unevaluated or undiagnosed abdominal pain may be rewarding and is essential to forfend life-threatening complications like perforative peritonitis.

Type II intramesenteric small bowel duplications, duplications sharing a wall or lumen with the adjacent bowel require a simultaneous resection and anastomosis of the adjacent segment of bowel. For isolated enteric duplications, like in our case, with a separate mesenteric pedicle, resection of the duplication cyst and the mesenteric pedicle is appropriate. Postoperative outcomes are generally favorable.

## Conclusion

Enteric duplication cyst is a rare pathology with a clinical spectrum that ranges from asymptomatic to perforative peritonitis. A high index of suspicion is required for diagnosis. It is important to be aware that common presentations in the emergency room like intestinal obstruction, perforative peritonitis, and complicated appendicitis may be manifestations of an underlying enteric duplication cyst when another more common source cannot be identified. The management in incidental presentations continues to be debated. However, in our opinion, surgical resection is imperative in symptomatic patients to avoid complications like obstruction, recurrent GI bleed, and perforative peritonitis.
